# Pharmacokinetic Profile of Doxycycline in Koala Plasma after Weekly Subcutaneous Injections for the Treatment of Chlamydiosis

**DOI:** 10.3390/ani12030250

**Published:** 2022-01-20

**Authors:** Chien-Jung Chen, Amber Gillett, Rosemary Booth, Benjamin Kimble, Merran Govendir

**Affiliations:** 1 Sydney School of Veterinary Science, The University of Sydney, Sydney, NSW 2006, Australia; benjamin.kimble@sydney.edu.au (B.K.); merran.govendir@sydney.edu.au (M.G.); 2 Australia Zoo Wildlife Hospital, Beerwah, QLD 4519, Australia; amber@wildlifewarriors.org.au (A.G.); rosemarybooth1@bigpond.com (R.B.)

**Keywords:** koalas, doxycycline, pharmacokinetics, chlamydiosis, *Phascolarctos cinereus*, HPLC

## Abstract

**Simple Summary:**

Doxycycline is an antimicrobial used for treating chlamydial infections in various species, including the koala. The dose and route of administration used initially are based on first principles. Therefore, this study investigates the absorption, distribution, metabolism, and excretion of subcutaneous doxycycline injections, and evaluates the suitability of the current dosage regimen for inhibiting chlamydial pathogens. The results suggest that the current doxycycline dosage remained therapeutically effective for up to six days after each dose, with some accumulation over successive doses. All koalas in the study improved clinically and tested negative for chlamydial pathogens post-treatment before being released. This study contributes to determining the optimal dosage of doxycycline to treat chlamydiosis safely and effectively in infected koalas.

**Abstract:**

Six mature, male koalas (*Phascolarctos cinereus*), with clinical signs of chlamydiosis, were administered doxycycline as a 5 mg/kg subcutaneous injection, once a week for four weeks. Blood was collected at standardised time points (T = 0 to 672 h) to quantify the plasma doxycycline concentrations through high-pressure liquid chromatography (HPLC). In five koalas, the doxycycline plasma concentration over the first 48 h appeared to have two distinct elimination gradients; therefore, a two-compartmental analysis was undertaken to describe the pharmacokinetic (PK) profile. The average ± SD maximum plasma concentration (C_max_) was 312.30 ± 107.74 ng/mL, while the average time ± SD taken to reach the maximum plasma concentration (T_max_) was 1.68 ± 1.49 h. The mean ± SD half-life of the distribution phase (T_1/2_ α) and the elimination phase (T_1/2_ β) were 10.51 ± 7.15 h and 82.93 ± 37.76 h, respectively. The average ± SD percentage of doxycycline binding to koala plasma protein was 83.65 ± 4.03% at three different concentrations, with a mean unbound fraction (*fu*) of 0.16. Using probability of target attainment modelling, doxycycline plasma concentrations were likely to inhibit 90% of pathogens with the doxycycline minimum inhibitory concentration (MIC) of 8.0–31.0 ng/mL, and the reported doxycycline MIC to inhibit *Chlamydia pecorum* isolates at the area under the curve/minimum inhibitory concentration (AUC/MIC) target of ≥24. All koalas were confirmed to be negative for *Chlamydia pecorum* using loop-mediated isothermal amplification (LAMP), from ocular and penile urethra swabs, three weeks after the last doxycycline injection.

## 1. Introduction

Chlamydiosis, predominantly caused by *Chlamydia pecorum*, is the most significant infectious disease affecting wild koalas, with 52% of koalas in South-East Queensland (SEQ) displaying clinical signs suggestive of chlamydiosis, and an infectious prevalence of up to 100% in some populations [1,2]. Common clinical signs in koalas associated with chlamydial infection include conjunctivitis leading to blindness [2], and/or inflammation of the urogenital tract causing cystitis, both of which are painful and can result in significant morbidity and mortality [1]. Infection can also lead to infertility through the development of paraovarian bursal cysts in females [3] and pathological changes in male reproductive organs [4].

The investigation of an alternative systemic administration route is warranted, as increased risk has been associated with doxycycline treatments administered orally to the koala, resulting in the disruption of gut microbiome and consequent dysbiosis [5]. The administration of once-weekly subcutaneous (s.c.) doxycycline injections at 5 mg/kg has been recently recommended as a treatment option for chlamydiosis in koalas [6,7], but the pharmacokinetic (PK) profile of doxycycline in the koala has not been reported for this route of administration. Therefore, the aim of this study was to describe the PK profile of doxycycline when administered subcutaneously to koalas at this dosage regimen, and to evaluate the suitability of this dosage regimen to inhibit chlamydial pathogens.

## 2. Materials and Methods

### 2.1. Animals and Housing

This study was approved by The University of Sydney Animal Ethics Committee protocol 2018/1360.

Wild koalas with disease or trauma are frequently presented to the Australia Zoo Wildlife Hospital (AZWH) at Beerwah for treatment. Six mature males admitted to AZWH with clinical signs indicative of chlamydiosis were recruited for this study. Animals were also selected if they had a good demeanour (to aid regular blood collections), mild to moderate clinical signs and were in reasonable health, and did not have pouch young. Incidentally, the study population were all males. The koalas’ bodyweight, body condition score, and age, as determined by the wear on the premolar and molar teeth [8], were recorded and are presented in Table 1. During this study, the koalas were housed individually in enclosures and supplied daily with fresh eucalypt browse and water ad libitum.

### 2.2. Clinical Examination and Blood Collection

Each koala underwent a clinical examination under general anaesthetic via an intramuscular (*i.m.*) injection of 3 mg/kg alphaxalone (Alfaxan^®^-CD, RTU; Jurox Pty Ltd., Rutherford, Australia) and then maintained with 2% isoflurane in 100% oxygen delivered via a face mask or cuffed 4–4.5 mm endotracheal tube. Overt signs of chlamydial disease where present were noted, and included: the degree of chemosis, conjunctiva proliferation, blepharospasm, epiphora and corneal opacity, and the presence of a wet or stained rump. Ultrasonography was used to assess the degree of pathology of the kidneys and the reproductive tract (prostate in males), and to measure bladder wall thickness. Chlamydial infection was confirmed via loop-mediated isothermal amplification (LAMP) positive swabs of the conjunctivae and penile urethra. Although the polymerase chain reaction (PCR) method is considered to be the ‘golden standard’ for its high sensitivity and specificity, LAMP is used to achieve rapid pathogen diagnosis in-house, which is critical to prevent delays in diagnosis and treatment, and is also reported to have high sensitivity and specificity in detecting *C. pecorum* in koalas [9].

An indwelling 20-gauge, 1 ¼-inch intravenous catheter was inserted into the cephalic vein and bandaged in place for blood collection up to 48–96 h depending on whether it remained necessary. Thereafter, blood was collected via venipuncture from the cephalic vein with a 23 g needle.

Each koala was administered a weekly subcutaneous doxycycline oil-based injection at 5 mg/kg (Vetafarm, Wagga Wagga, Australia), diluted at 50:50 in saline, for 4 weeks. Serial blood samples of 2 mL were collected from the cephalic vein into sodium heparin tubes before treatment (T = 0 h), as well at T = 1, 2, 4, 8, 12, 24, 48, 96, 144, 168, 336, 504, and 672 h for all koalas, except for K2, with only 13 time points (T = 0, 1, 2, 4, 8, 12, 24, 72, 120, 168, 336, 504 and 672 h).

In addition to doxycycline, koalas with evidence of significant inflammation of the conjunctiva and bladder mucosa were treated with other medications, as reported in Table 1. Koalas with conjunctivitis were treated with topical chloramphenicol and hydrocortisone acetate eye ointment (Chloroptosone^®^, Ceva Animal Health Pty Ltd., Glenorie, Australia), or ofloxacin eye drops (Ocuflox^®^ eyedrops, Allergan Australia Pty Ltd., Gordon, Australia) for at least the first week. K3 was also administered dexamethasone eye drops (Maxidex^®^, Novartis Pharma Australia Pty Ltd., Macquarie Park, Australia) once only at the beginning of the first doxycycline injection. Prednisolone, used as an anti-inflammatory, was administered to koalas (K1 and K2) showing discomfort during urination. K5 was also medicated with an oral antifungal nystatin (Nilstat^®^ oral drops, Aspen Pharma Pty Ltd., St Leonards, Australia) to treat gastrointestinal candidiasis prior to the fourth doxycycline injection. Koalas K2, K3, and K5 were administered intravenous (i.v.) or s.c. fluids (compound sodium lactate (Hartmann’s)) for a few days at various times during the doxycycline treatment regime to correct dehydration.

### 2.3. Pooled Blank Plasma

Blank koala plasma was pooled from clinically normal koalas from Taronga Zoo (Mosman, Australia) (*n* = 5) and was stored at −20 °C before thawing at room temperature to prepare standards of known drug concentrations and to determine the mechanism of doxycycline binding to plasma proteins (PPB). The blank koala plasma was retrieved opportunistically as approved by the Taronga Zoo Conservation Society, protocol 3b/04/19.

Blood samples were centrifuged at 6000 rpm for 10 min within 1 h of collection, and the plasma was transferred to plain tubes. The plasma was stored at −80 °C before transportation from AZWH to The University of Sydney on dry ice. All samples were analysed within one month of sample collection.

### 2.4. Drug Analysis

Doxycycline hyclate and tetracycline, as the internal standard (IS), were purchased from Sigma Aldrich (Castle Hill, Australia). Ammonium acetate, ethylenediaminetetraacetic acid (EDTA), magnesium chloride, triethylamine, acetonitrile, and methanol were purchased from Thermo Fisher Scientific (Macquarie Park, Australia). Purified water was retrieved from the Milli-Q water purification system (Merck Millipore, Burlington, MA, USA).

Reversed-phase, high-pressure liquid chromatography (HPLC) was modified from previous methods [10,11] using fluorescence detection to suit the complexity of the koala plasma. The ultra-performance liquid chromatography (UPLC) system (Shimadzu, Rydalmere, Australia) included an SCL-40 system controller, a DGU-405 degassing unit, an LC-40D XR solvent delivery module, an SIL-40C XR auto sampler, an RF-20A XS fluorescence detector, and a CTO-40C column oven.

Chromatographic separation was performed using an Apollo C_18_, 5 µm, 250 mm × 4.6 mm (Grace, Columbia, MD, USA) with the column temperature maintained at 40 °C. The isocratic mobile phase was composed of acetonitrile, methanol, and buffer (50 mM ammonium acetate, 2 mM EDTA, 50 mM magnesium chloride, 0.3% triethylamine in purified water) (10:7:83, *v*/*v*/*v*), adjusted to pH 7.4 with galactic acid. The mobile phase was delivered at a flow rate of 1.2 mL/min, with the fluorescence detector operating at an excitation wavelength of 380 nm and an emission wavelength of 520 nm. The total run time for each sample was 20 min.

### 2.5. Sample Preparation

New standards were freshly prepared for each day of analysis using pooled koala plasma obtained from clinically normal koalas from Taronga Zoo. Plasma samples were spiked with a doxycycline stock solution (0.5 mg/mL) to obtain standards of 7.81, 15.63, 31.25, 62.5, 125, 250, 500, and 1000 ng/mL. A stock solution of the IS (0.5 mg/mL tetracycline) was prepared in acetonitrile and was further diluted with acetonitrile to give a working solution of 2500 ng/mL. The IS stock solution was stored at 3 °C and the working solution was freshly prepared as required. The pooled blank plasma and doxycycline stock solution were also used to prepare the quality control (QC) samples of low, medium, and high concentrations (7.81, 125, and 1000 ng/mL) for assay validation.

Protein precipitation was used as the drug extraction method from the plasma. The process involved adding 5 µL of trichloroacetic acid (2 g/mL TCA in water), 200 µL of acetonitrile with 10 µL of IS working solution into 200 µL of each plasma standard. The sample was vortexed and then centrifuged at 14,000 rpm for 10 min. Then, 100 µL of supernatant was mixed with 400 µL of mobile phase in a separate 1.5 mL Eppendorf^®^ tube. The sample was vortexed and then centrifuged at 14,000 rpm for 10 min again, before injecting 20 µL of the supernatant for HPLC analysis. This was repeated twice (for a total of three times) for each standard and QC sample. Samples were stored at −20 °C away from light before thawing at room temperature for analysis.

### 2.6. Assay Validation

The validation of the analytical conditions consisted of determining the specificity and sensitivity, linearity, range, accuracy, precision, lower limit of detection (LLOD), and lower limit of quantitation (LLOQ) of the assay [12]. The specificity was determined by identifying the peaks and retention times of doxycycline and the IS. Consistent tetracycline and doxycycline peaks were observed in the QC samples with the retention times of ≈6.5 min and ≈9.5 min, respectively. The linearity and sensitivity were established by analysing the standard doxycycline concentrations to generate a standard curve on each occasion. Linearity was determined by the linear regression of the calibration curve (y = ax + b), while the sensitivity was based on the LLOD and the LLOQ values. A weighting factor (1/x) was applied to ensure that the observations were correctly fitted, especially at the lower concentrations. The LLOD and LLOQ were calculated using the formulae [13]:

LLOD=(σ/X) × 3, LLOQ=(σ/X) × 10.

where σ = the standard deviation of the *y*-axis intercepts and X = the average of the standard curve gradients.

The computation of intra- and inter-day assay accuracy and precision used the same QC concentrations. Accuracy (%) was calculated by dividing the estimated concentration by the actual concentration multiplied by 100, while the precision (CV, %) was obtained by dividing the standard deviation by the mean estimated concentration, multiplied by 100 [14]. The cut-off precision and accuracy of the calculated values are required to be within ±20% of the expected values [14].

The stability of doxycycline in the koala plasma was established by spiking the pooled blank plasma with doxycycline (1000 ng/mL), and then storing it at −20 °C over 3 months. Working solutions of 1000 ng/mL were freshly prepared on the day of analysis every month in triplicates, following the method described.

Drug recovery was determined by ‘spiking’ doxycycline into blank koala plasma to achieve concentrations of 7.81, 125, and 1000 ng/mL, while the concentration of the IS remained at 125 ng/mL. Another batch was prepared in Milli-Q water, instead of blank koala plasma, and spiked with identical doxycycline and IS concentrations. All samples in the plasma and water were analysed as triplicates. The samples were evaluated to obtain the areas of the doxycycline peaks after following the sample preparation and the protein precipitation extraction methods. The average drug recovery percentage at each concentration was calculated by:

$$\%\;\mathrm{of}\;\mathrm{average}\;\mathrm{drug}\;\mathrm{recovery}=\frac{\mathrm{Mean}\;\mathrm{doxycycline}\;\mathrm{peak}\;\mathrm{area}\;\mathrm{in}\;\mathrm{plasma}}{\mathrm{Mean}\;\mathrm{doxycycline}\;\mathrm{peak}\;\mathrm{area}\;\mathrm{in}\;\mathrm{water}}\;\times \;100$$

### 2.7. Pharmacokinetic (PK) Analysis

Compartmental models were chosen to determine the PK parameters and indices using PKSolver [15]. Five of the six koalas were analysed with a two-compartmental model and the other (K5) with a one-compartmental analysis. Both the T_max_ and C_max_ were obtained visually from the concentration in plasma vs. time semi-log graph. The drug elimination constant (k_el_) was retrieved by estimating the semi-log linear regression of the terminal slope, while the t_1/2_ was calculated with the Equation [16]:

$$T_{1/2}=\frac{\ln\;(2)}{k_{\mathrm{el}}\;}$$

The linear-log trapezoidal method was used to calculate the area under the curves (AUC_0–t_) and the area under the first moment curve (AUMC_0–t_) from 0 to 168 h (over the first 7 days). The equations used for the AUC and AUMC from the observed concentration to infinity were [17]:

$${\mathrm{AUC}}_{t–\infty }=\frac{\mathrm{Clast}}{k_{\mathrm{el}}\;}$$

$${\mathrm{AUMC}}_{t–\infty }=(\frac{{\mathrm{Clast}\;\times \;T}_{\mathrm{last}}\;}{k_{\mathrm{el}}\;})+\frac{C_{\mathrm{last}}}{{k_{\mathrm{el}}}^{2}}$$

As the bioavailability (F) is unknown when administered subcutaneously, the mean residence time (MRT), apparent clearance (Cl_app_), and apparent volume of distribution associated with the elimination phase (V_area_ or V_z_) were calculated using the following formulae [18]:

$$\mathrm{MRT}=\frac{\mathrm{AUMC}\;}{\mathrm{AUC}}$$

$$\mathrm{Clapp}=\frac{\mathrm{Cl}\;}{F}\;\mathrm{where}\;\mathrm{Cl}=\frac{\mathrm{Dosesc}\;}{\mathrm{AUCsc}}$$

$$\mathrm{Vz}=\frac{\mathrm{Cl}\;}{\mathrm{kel}}$$

Calculations were also undertaken to ascertain whether the doxycycline was accumulating in the trough concentrations prior to the administration of each dose. The accumulation factor expresses the number of times the concentration in the plasma will be higher after *n* doses compared to the first dose, using the formula [16,19]:

$$\mathrm{Accumulation}\;\mathrm{factor}=\frac{(1\;-{\;e}^{-\mathrm{nk}\tau })}{(1\;-{\;e}^{-k\tau })}$$

$$k=\frac{\ln\;(2)}{T_{1/2}\;\beta \;}$$

where k = the elimination rate constant, *n* = the number of doses, τ = the dosage interval (h), and T_1/2_β = the rapidity of the elimination phase after drug distribution equilibrium.

### 2.8. Doxycycline Binding to Koala Plasma Proteins (PPB)

The doxycycline binding to plasma procedure was modified based on the rapid equilibrium dialysis (RED) method for the measurement of the doxycycline unbound fraction (*fu*) in the koala plasma [20]. The pooled koala plasma was spiked with doxycycline to produce three different concentrations of 7.81, 62.5, and 1000 ng/mL and adjusted to pH 7.4, with the analysis performed as duplicates. For each concentration, 200 µL of pre-spiked plasma was pipetted into the sample (red-ringed) chamber, and 400 µL of phosphate buffered saline (PBS) (100 mM sodium phosphate and 150 mM sodium chloride, adjusted to pH 7.4) was added to the buffer (white-ringed) chamber of the RED device, as per the manufacturer’s recommendation (Thermo Fisher Scientific, Macquarie Park, Australia). Validation samples were also prepared by adding 200 µL of doxycycline (1000 ng/mL) with PBS into the sample chamber and 400 µL of PBS into the other. The RED devices were incubated at conditions of 200 rpm at 37 °C for 3 h. Then, 100 µL was removed from the sample chamber to undergo sample preparation (method described previously) after incubation, then spiked with 10 µL of internal standard (125 ng/mL tetracycline). Finally, 100 µL of supernatant from the sample chamber and 100 µL from the buffer chamber were extracted for HPLC analysis.

### 2.9. Probability of Target Attainment (PTA) and Monte Carlo Simulation

The family of tetracyclines are considered to be concentration-dependent antibacterial drugs [21]. Therefore, the clinical efficacy of doxycycline for inhibiting a specific bacterial pathogen can be determined by the ratio of the AUC over 24 h to the minimum inhibitory concentration (MIC) (AUC_0–24_/MIC) [22], with a suggested efficacious pharmacokinetic–pharmacodynamic (PK/PD) target of ≥24 in dogs [23,24,25], according to the following Equation [26]:

$$\mathrm{Dose}=\frac{\frac{\mathrm{CL}}{F}\times \left(\frac{\mathrm{AUC}}{\mathrm{MIC}}\right)\times \;\mathrm{MIC}}{fu\;\times \;F\;\times \;24\;h}$$

The PK/PD indices were calculated using the equation below, which was derived from the equation above for determining a maintenance dose at a specific AUC/MIC target [27]:

$$\frac{\mathrm{AUC}}{\mathrm{MIC}}\;\geq \;24=\frac{\mathrm{Dose}\;\times \;fu}{\mathrm{CL}\;\times \;\mathrm{MIC}}$$

where the AUC/MIC = the area under the curve divided by the minimum inhibitory concentration over 24 h, MIC = the targeted pathogen, dose = the average dose (µg), CL/F = the average apparent clearance, *fu* = the fraction unbound, and F = the bioavailability factor.

PTA analysis also incorporated the between-subject variation of the total dosage (weight × dose) and the apparent clearance.

The PK/PD indices obtained from the calculations, along with nine MICs of 0.008, 0.01, 0.03, 0.06, 0.1, 0.12, 0.25, 0.5, and 1.0 µg/mL, were entered into a forecasting program (Crystal Ball^®^, Oracle Software, Denver, CO, USA). A Monte Carlo simulation, generated for 1000 trials, was repeated three times for each MIC to yield the mean PTAs (%).

## 3. Results

### 3.1. Summary of the Koalas

The signalment and treatment summary of the six male koalas (K1–6), are provided in Table 1. All koalas had haematology and biochemical analyte values within the normal ranges for this species [28,29].

### 3.2. Assay Validation

The stability of doxycycline in koala plasma was evaluated over three months, where doxycycline was stable until the third month (22.56% degraded from the previous month) when the sample was stored at −20 °C away from light. Therefore, the stability test was discontinued on the third month due to the significant doxycycline degradation observed.

The doxycycline recovery, obtained from concentrations of 7.81, 125, and 1000 ng/mL, were 82.54 ± 4.63%, 90.04 ± 1.69%, and 88.48 ± 1.21%, respectively, with the average ± standard error of mean (SEM) doxycycline recovery of 87.02 ± 3.23% across the three concentrations. The recovery of the IS was calculated as 95.84 ± 4.13%.

The estimated doxycycline concentrations of the intra- and inter-day QC samples (mean ± SD) dosed with 125 and 1000 ng/mL, and their accuracy and precision, are provided in Table 2.

The validated and optimised condition, calculated using the formulae described, yielded LLOD and LLOQ values of 1.57 and 5.24 ng/mL, respectively.

### 3.3. PK Analysis

**Figure 1:**
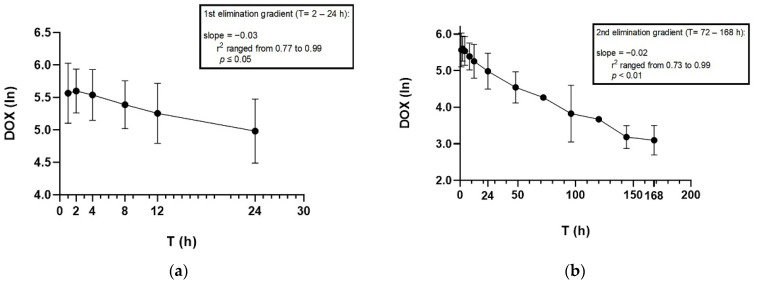
Semi-log graphs showing the mean ± SD of doxycycline plasma concentration when koalas are administered doxycycline 5 mg/kg by the first subcutaneous injection (diluted 50:50 with saline) over (**a**) 24 h and (**b**) 168 h.

Graphical representations of the change in doxycycline plasma concentrations (log scale) over time are provided in Figure 1a for 0–24 h and Figure 1b for 0–168 h (7 days). The semi-log graphs of the mean doxycycline concentrations in plasma of five koalas portrayed a distribution and an elimination gradient, suggesting a two-compartment model, which was further supported by its Akaike information criterion (AIC) model of fit [30]. The analysis was weighted for all koalas based on a weighting factor of 1/C^2^.

Linear regression was used to calculate the first elimination gradient from 2 to 24 h and the second elimination gradient from 72 to 168 h.

The mean, median, and range of the doxycycline concentrations in the plasma for the koalas at various time points, with the calculated PK/PD ratios using median AUC values, are presented in Table 3. The individual doxycycline plasma concentrations for each koala at each time point are available in Table S1.

The relevant pharmacokinetic parameters and indices of doxycycline (mean ± SD and median) are presented in Table 4.

Doxycycline concentrations in the plasma and the accumulation ratio at the trough concentrations of dose 1 (T = 168 h), dose 2 (T = 336 h), and dose 3 (T = 672 h) are provided in Table 5.

**Figure 2:**
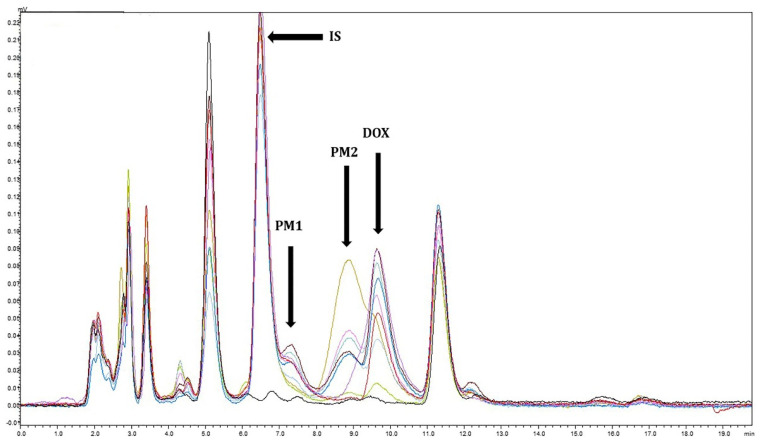
Chromatograms of K3’s plasma 1 h and 672 h after administration of doxycycline at 5 mg/kg via subcutaneous injection (diluted at 50:50 with saline), once a week for four weeks. Each line represents the koala plasma at different time points (T = 0 h (black) to T = 144 h (light green)). Possible metabolites are PM1 and PM2; DOX = doxycycline; IS = internal standard.

Interferences with the doxycycline peak, possibly due to potential metabolites, which were observed in the plasma of a few koalas without other additional antibiotics administered, are demonstrated in Figure 2.

### 3.4. Plasma Protein Binding

Doxycycline is known to have high an affinity for plasma proteins, as the yielded mean ± SD doxycycline PPB (%) in koalas at concentrations of 7.81, 62.5, and 1000 ng/mL were 92.56 ± 6.90, 85.37 ± 4.77, and 73.01 ± 0.43%, respectively. The average doxycycline plasma protein binding in koalas yielded 83.65 ± 4.03% across the three concentrations, with the mean unbound fraction of 0.16 (Table 6).

### 3.5. Probability of Target Attainment

**Figure 3:**
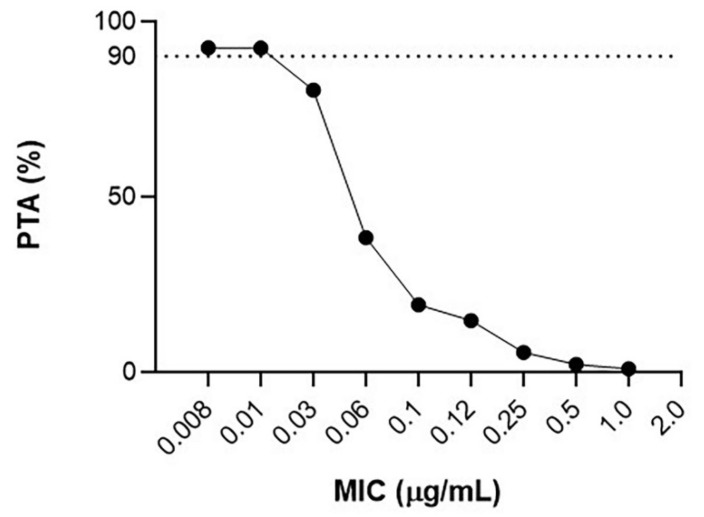
Probability of target attainment (≥90%) using a Monte Carlo simulation with 1000 hypothetical trials, when doxycycline at 5 mg/kg is administered via subcutaneous injection (diluted at 50:50 with saline), at serial doubling doxycycline MICs for AUC/MIC target ≥24.

The PTA of doxycycline against *C. pecorum*, given the current treatment regimen, at doxycycline MICs from 0.008 to 1.0 µg/mL for the AUC/MIC target of ≥24 is presented in Figure 3.

## 4. Discussion

This is the first study to document the PK profile of doxycycline in free-ranging koalas with chlamydiosis. As a concentration-dependent antibiotic, the efficacy of doxycycline at inhibiting pathogens is based on the PK/PD relationship of the unbound fraction x AUC_24_/MIC ≥ 24 [31], which has been agreed on for doxycycline in humans [22], mice [32], and dogs [23]. This target was used to predict the efficacy for doxycycline when administered at 5 mg/kg, once a week for four weeks, for treating *C. pecorum*.

Even though this study did not investigate the doxycycline MIC for inhibiting *C. pecorum*, reported as 8.0–31.0 ng/mL [33], the proportion of doxycycline binding to plasma proteins to provide the unbound fraction was explored. Therefore, according to the PK/PD relationship discussed above, the current dosage suggests that doxycycline remains therapeutically effective for the duration of up to two, three, and even six days after each dose, with the median PK/PD ratios of 42.63, 31.73, and 35.36 at the respective time points at MICs of 31.0, 20.0, and 8.0 ng/mL (Table 3). From the PTA analysis, this treatment was found to be efficacious (PTA of ≥90%) against pathogens with a doxycycline MIC ≤ 0.01 µg/mL (or 10.0 ng/mL) (Figure 3) and therefore, using the doxycycline AUC of 0–24 h, this dosage of doxycycline should inhibit 90% of intravascular bacterial pathogens with a doxycycline MIC range mentioned previously.

Chlamydial pathogens are intracellular and, although antibiotic plasma concentrations are considered to be a surrogate for intracellular concentrations [34,35], doxycycline may accumulate intracellularly. All animals clinically improved over the four weeks of treatment and tested chlamydia negative according to a LAMP test post-treatment, suggesting that doxycycline reached efficacious concentrations at this dosage with some accumulation over successive doses (Table 5). However, this is only considered to be weak accumulation (1.2 ≤ accumulation factor < 2) [36], so toxicity due to drug accumulation is unlikely for this dosage regimen, but this may vary between animals.

The semi-log plots of the doxycycline concentration in plasma of the koalas at each time point suggest that compartmental analysis was more suitable than non-compartmental analysis (Figure 1). Following the comparison of one-compartmental and two-compartmental analyses, the pharmacokinetic parameters and indices of doxycycline for K1–K4 and K6 were calculated with the two-compartmental model, while K5 was analysed with a one-compartmental model, which was mainly determined by the lowest AIC value indicating a better fit (Table 4) [30]. A two-compartment analysis was also the best-fitting PK model in other species, such as dogs [37,38], cats [37], chickens [39], horses [40], sheep [41], pigs [42], and turkeys [43].

The mean ± SD half-life of the drug distribution phase (T_1/2_ α) was faster than that of the elimination phase (T_1/2_ β), at 10.51 ± 7.15 h and 82.93 ± 37.76 h, respectively. This is similar to the doxycycline disposition in dogs, with a T_1/2_ α of 9.3 h and T_1/2_ β of 133.61 h [38], and could be an indicator that doxycycline distributes at a similar rate, but with a faster terminal elimination in the koala (Table 4). Although the mean residence time (MRT) has been determined in other species, there are no studies that were administered at 5 mg/kg subcutaneously to compare to that in the koala.

Importantly, the apparent volume of distribution (V_z_/F) is high at 16.60 ± 6.75 L/kg, meaning doxycycline is mainly in the extravascular compartments, such as tissues, rather than plasma (Table 4). Although there are no published data to directly compare the apparent clearance (Cl/F) of subcutaneously administered doxycycline in other species, the Cl/F value of 0.35 ± 0.15 L/kg/h obtained for koalas is noticeably faster than the doxycycline clearance in other species administered intravenously or intramuscularly, including dogs at 0.13 ± 0.01 L/kg/h [38], cats at 0.07 ± 0.01 L/kg/h [37], goats at 5.72 × 10^−4^ ± 1.23 × 10^−4^ L/kg/h [44], and chickens at 0.10 ± 0.02 L/kg/h [39].

Although identical doxycycline dosages were administered subcutaneously across all six koalas, the doxycycline concentration in K6’s plasma was noticeably higher than the others, especially within the first two hours of injection (T = 1 h and T = 2 h) at 498.89 and 474.38 ng/mL, respectively (Table S1). Variation in the drug plasma concentrations between koalas are also reported in other studies [45,46,47]. Possible factors could include underlying illness conditions (e.g., fever and hypotension), and variations of diet and metabolism of the animal. However, K6’s plasma doxycycline concentration at the end of the first seven days (T = 168 h) was similar to the others at 27.06 ng/mL.

The unbound fraction is considered to have a pharmacologic effect; hence, a higher unbound fraction may result in greater drug activity [48]. The unbound fraction of doxycycline in koala plasma is two times higher than in dogs at 0.08 [49]. In comparison, the unbound fraction of humans and horses are similar to that of koalas at 0.1 to 0.2 [50] and 0.19 [51], respectively (Table 6).

**Table 6:** The unbound fraction (*fu*) of doxycycline when interacting with koala plasma proteins, and the plasma proteins of other species using various methods.

Species	Method	Fraction Unbound (*fu*)	References
Koalas (conducted in duplicates at each concentration)	In Vitro rapid equilibrium dialysis	1000 ng/mL: 0.27; 62.5 ng/mL: 0.15; 7.81 ng/mL: 0.07; Mean: 0.16	This study
Dogs	In Vitro ultrafiltration	0.08	[49]
Humans	Not specified	0.1–0.2	[50]
Horses	In Vitro ultrafiltration	0.19	[51]

However, no conclusions can be made, as doxycycline was administered orally at much greater dosages in those studies. As recently collected (or fresh) koala plasma samples were not readily accessible, the plasma was previously frozen and thawed on the day of analysis. Using thawed frozen plasma can be a limitation when determining the drug’s binding to plasma proteins, as the pH of frozen plasma may differ from that of fresh plasma, which may affect the binding of drugs to proteins [52,53]. A study compared the protein binding of sulfamethazine in fresh and frozen plasma, and reported more binding in fresh plasma as the drug concentration increases [54]. The protein binding of doxycycline is pH dependent, so the pH was adjusted to that of the fresh plasma (pH 7.4) [55].

Validations of the doxycycline assay were based on the intra- and inter-day analysis of the QC samples, with the averages recording accuracies and precisions within the 20% bias, with a few exceptions outside that range by <1% (Table 2). Validation data for each day are available in Table S3. In comparison to the fluorescence detection methods reported in other species, this assay had comparable selectivity and sensitivity (LLOD = 1.57 ng/mL; LLOQ = 5.24 ng/mL), with the LLOD and LLOQ values of 1.63 ng/mL and 4.93 ng/mL in human gingival crevicular fluid, 6.36 ng/mL and 19.28 ng/mL in human saliva [10], an LLOD value of 1.2 ng/mL in turkey liver, and an LLOD value of 1.0 ng/mL in turkey muscle tissues [11]. However, as the mean and median doxycycline concentrations at T = 672 h are questionable compared to the previous time points, the LLOQ may realistically be closer to 20 ng/mL.

There were some interferences observed in the chromatograms from T = 4 h onwards, more noticeably in K3, which were not detected in the blank plasma (T = 0 h). K3, on initial treatment, had conjunctivitis, so ocular medications (chloramphenicol, prednisolone, and dexamethasone eye ointments) were applied to both eyes. Although the HPLC assay condition was selective for doxycycline, it is also possible that chloramphenicol and hydrocortisone acetate were detected in the plasma, causing the interferences. Further, as the early time point samples were collected during short general anaesthesia, anaesthetic drugs may also interfere if detected in the chromatograms. However, alphaxalone was not detectable using the HPLC condition. Another possible reason could be due to potential metabolites, as the peaks of PM1 and PM2 gradually increased, then decreased over time (Figure 2), and were absent in the blank and spiked plasma samples.

Limitations in this study included: (a) the use of plasma concentrations instead of intracellular concentrations; (b) only koalas with active chlamydial infections were investigated; (c) the use of the accepted PTA target of 24; (d) limited sample size; and (e) the use of drugs other than doxycycline. As *C. pecorum* is an intracellular bacterial pathogen, the intracellular doxycycline concentration may be greater than the plasma concentration, with the plasma concentration being used when determining the efficacy of the treatment. Drugs’ PK parameters can vary among clinically healthy and infected animals, which is evident in humans with renal disease, where a significant reduction in protein binding [56] and an increase in AUCs [57] were observed. Therefore, without understanding the doxycycline PK profile in healthy koalas, whether dose modification is required for animals with other health conditions remains uncertain. While the PTA was based on doxycycline plasma concentrations, doxycycline has been reported to penetrate tissues effectively [50], so the PTA of ≥24 may be too high for the inhibition of intracellular pathogens. Another limitation was that the study used the only published MIC range of *C. pecorum* for doxycycline with only three isolates tested [33]. Importantly, as the oil-based doxycycline injections were used in this study, the PK profile may differ when comparing them to the more aqueous doxycycline injections from other manufacturers. Other limitations include a small sample size and only male involvement. The sample size is usually low in pharmacology studies in this species, due to the high perceived value of each individual animal [58,59]. However, the information obtained here provides valuable data on this drug in this species and will be a foundation for future data to support the many observations described in this study. Lastly, some of the auxiliary antibiotics, anti-inflammatories, or analgesics used may have an inhibitory or induction effect of some drug metabolism enzymes. Chloramphenicol is an inhibitor of cytochrome (CYP) P450 2C19 and CYP3A4 [60], but as it was applied topically, the drug concentration in the plasma would be minimal. Although the metabolic pathways of doxycycline are unidentified, there is no evidence that systemically administered prednisolone or nystatin should affect the rate of elimination of doxycycline.

Reported side effects in koalas treated with doxycycline include the occasional weight loss, depression, candidiasis, dysbiosis, and typhlocolitis [7]. The onset and severity of these side effects appears to vary considerably between individuals. Koalas in this study experienced some side effects during and shortly after treatment. For example, weight loss and the development of candidiasis were observed in K5, and a decrease in body condition and dehydration requiring IV fluid therapy were experienced in K2 and K6. Doxycycline injections can be painful due to the increased viscosity of the formulation; however, diluting in saline and administering subcutaneously reduces the pain [7], and weekly administration is an advantage in frightened and aggressive wild animals.

## 5. Conclusions

This study successfully described the PK profile of doxycycline in koala plasma and increased the confidence of the treatment’s effectiveness when administering doxycycline subcutaneous injections (once per week for 4 weeks) at 5 mg/kg. Considering the data analysis and the outcome of the animals in this study, further studies could consider reducing the dosage and duration, which may raise questions regarding the suitability of using PK/PD target of ≥24 in koalas and possibly reduce the severity of the known side effects. Additionally, investigating the potential doxycycline metabolites, and their activity, would provide more insights into the therapeutic effects. While determining the PK profile from plasma concentrations is a start, using intracellular concentrations would be more relevant for understanding drug activities in tissues. This study contributes to determining the optimal dosage to treat chlamydiosis safely and effectively in all infected koalas.

## Figures and Tables

**Table 1:** Demographic information of the six male koalas recruited and their axillary drug treatments during the administration of doxycycline 5 mg/kg by subcutaneous injection (diluted 50:50 with saline), once a week for four weeks.

Koala	Age (years)	Weight (kg)	Body Condition Score	Clinical Sign/s	LAMP Result (Pre-Treatment)	Drugs Administered	Clinical Sign/s Resolved?	LAMP Result (Post-Treatment)	Outcome
K1	3	6.3	6/10	Cystitis	Positive (UGT)	Doxycycline, prednisolone 5 mg/kg PO SID from 48 h after blood collection for three doses then EOD for three further doses during doxycycline injections	Y	Negative	Released
K2	10	6.0	4/10	Cystitis	Positive (UGT, ocular)	IV fluids on admission for 24 h to correct dehydration. Doxycycline, prednisolone 5 mg/kg PO SID for three doses then EOD for seven further doses from day 12 for several days during doxycycline injections	Y	Negative	Released
K3	6	8.5	6/10	Conjunctivitis, cystitis	Positive (UGT, ocular)	*S.c.* fluids on admission to correct dehydration. Doxycycline, Chloroptsone administered twice daily for 14 days, and dexamethasone eye drops (once only)	Y	Negative	Released
K4	10	8.9	5/10	Cystitis	Positive (UGT, ocular)	Doxycycline	Y	Negative	Released
K5	5	7.7	6/10	Conjunctivitis	Positive (ocular)	Doxycycline, ofloxacin eye drops BID for the first 14 days. Repeat ofloxacin course BID with addition of dexamethasone BID for 14 days starting from the fourth doxycycline injection	Y	Negative	Released
K6	10	7.5	5/10	Conjunctivitis	Positive (UGT)	Doxycycline, IV fluids for 48 h from admission to correct dehydration after T = 0 h	Y	Negative	Released

Chloroptsone^®^ (chloramphenicol: 10 mg/g, hydrocortisone acetate: 5 mg/g topically). Post-treatment LAMP results were determined from swabs taken three weeks after the last doxycycline injection. LAMP = loop-mediated isothermal amplification; UGT = urogenital tract; Y = Yes; IV = intravenous; PO = oral administration; EOD = every other day; SID = once a day; BID = twice a day.

**Table 2:** Mean ± SD of the intra- and inter-day QC samples as triplicates dosed with doxycycline at different concentrations (125 and 1000 ng/mL), with the accuracy (%) and precision (%) of the intra-day and inter-day results across three days.

**Intra-Day (across 3 Days)**		
Expected concentration (ng/mL)	125	1000
Estimated concentration (ng/mL)	108.93 ± 4.46	1083.03 ± 68.52
Accuracy (%)	84.45–92.19	100.21–116.97
Precision (%)	4.10	6.33
**Inter-Day (across 3 Days)**		
Expected concentration (ng/mL)	125	1000
Estimated concentration (ng/mL)	115.71 ± 6.65	1068.06 ± 73.14
Accuracy (%)	88.01–96.03	104.31–110.78
Precision (%)	5.38	6.84

**Table 3:** Mean and standard deviation (SD), median, and range of the doxycycline plasma concentrations when koalas were administered doxycycline at 5 mg/kg via subcutaneous injection (diluted at 50:50 with saline), once a week for four weeks. Median PK/PD ratios of the six koalas with chlamydiosis at various time points over four weeks (672 h) are also provided.

Doxycycline Concentration in Plasma (ng/mL)					Median PK/PD		
T (h)	Mean	SD	Median	Range	MIC = 8 ng/mL	MIC = 20 ng/mL	MIC = 31 ng/mL
0	0	0	0	0	0	0	0
1	284.77	117.07	283.45	138.38–498.89	708.64	283.45	182.87
2	284.04	94.00	264.36	167.40–474.38	44.81	17.92	11.56
4	270.55	93.43	238.78	140.53–408.68	51.70	20.68	13.34
8	230.92	70.79	209.26	124.47–312.56	43.43	17.37	11.21
12	208.28	79.49	191.21	104.41–323.39	41.21	16.49	10.64
24	159.50	60.69	155.07	64.09–237.03	191.88	76.75	49.52
48	100.23 ^α^	32.14 ^α^	107.49 ^α^	47.23–140.61 ^α^	165.20	66.08	**42.63**
72	71.18 ^β^	0 ^β^	71.18 ^β^	N/A	79.32	**31.73**	20.47
96	55.38 ^α^	27.92 ^α^	48.70 ^α^	12.70–94.87 ^α^	27.19	10.88	7.02
120	39.25 ^β^	0 ^β^	39.25 ^β^	N/A	52.65	21.06	13.59
144	25.07 ^α^	6.81 ^α^	24.02 ^α^	15.10–36.38 ^α^	**35.36**	14.14	9.13
168	23.39	6.49	24.56	9.98–30.88	2.79	1.12	0.72
336	19.99	6.66	20.84	9.84–27.72	0.05	0.02	0.01
504	19.26	7.87	18.71	10.42–33.38	5.62	2.25	1.45
672	27.27	8.46	25.07	20.75–45.61	6.12	2.45	1.58

Bold indicates the last PK/PD ratio of ≥24. PK/PD ratio = unbound fraction × AUC_24_/MIC; ^α^ = data from five koalas; ^β^ = data from one koala; N/A = not available. PK uses median AUC and PD uses the MIC listed above.

**Table 4:** Mean ± SD, median, and range of the pharmacokinetic parameters and indices for doxycycline in koala plasma determined over the first seven days after administration of doxycycline at 5 mg/kg via subcutaneous injection (diluted at 50:50 with saline).

Parameters and Indices	Mean	SD	Median	Range
K_10_ (1/h)	0.02	0.004	0.02	0.02–0.03
K_12_ (1/h)	0.04	0.04	0.02	0.01–0.12
K_21_ (1/h)	0.06	0.04	0.07	0.01–0.10
T_1/2_ α (h)	10.51	7.15	6.50	2.97–22.67
T_1/2_ β (h)	82.93	37.76	64.25	46.02–137.87
T_1/2_ K_10_ (h)	-	-	-	-
T_max_ (h)	1.94	1.51	2.14	0.20–4.01
C_max_ (ng/mL)	324.67	114.07	345.86	155.64–504.24
AUC_0–t_ (ng/mL·h)	14,063.63	5099.77	15,887.91	5725.13–20,604.50
AUC_0–∞__obs (ng/mL·h)	16,295.38	5264.70	17,252.40	7765.74–22,464.07
AUC_0–t_/AUC_0–∞__obs	0.85	0.07	0.85	0.74–0.92
AUMC_o–∞__obs (ng/mL·h^2^)	1.37 × 10^6^	3.84 × 10^5^	1.16 × 10^6^	1.01 × 10^6^, −2.08 × 10^6^
MRT (h)	89.65	24.26	86.45	64.58–129.88
V_z_/F _obs (L/kg)	16.60	6.75	14.03	9.77–29.37
Cl/F_obs (L/kg/h)	0.35	0.15	0.29	0.22–0.64

K_10_ = first-order rate constant of drug elimination; K_12_ = rate constant for transfer of drug from compartment 1 to 2; K_21_ = rate constant for transfer of drug from compartment 2 to 1; T_1/2_ α = rapidity of the distribution phase after drug administration; T_1/2_ β = rapidity of the elimination phase after drug distribution equilibrium; T_1/2_ K_10_ = half-life of elimination of the free fraction; T_max_ = peak time; C_max_ = peak concentration; AUC_0–t_ = area under the plasma concentration–time curve from time of dosing to time of last measurable concentration; AUC_0-∞__obs = area under the plasma concentration–time curve from time of dosing to infinity; AUMC_o-∞__obs = area under the moment curve from time of dosing to infinity; MRT = mean residence time; V_z_/F_obs = apparent volume of distribution during terminal phase; Cl/F_obs = apparent total clearance; F = bioavailability; CV = coefficients of variation. All koalas were given a weighting factor of 1/C^2^. K5 was excluded from the mean, SD, median, and range.

**Table 5:** Mean ± SD, median, and range of trough doxycycline concentrations in plasma when koalas were administered doxycycline at 5 mg/kg via subcutaneous injection (diluted at 50:50 with saline) once a week for four weeks, and the accumulation factor prior to the 2nd, 3rd, and 4th dose.

	Doxycycline Concentrations in Plasma (ng/mL)		
Animal	Trough Concentration Prior to 2nd Dose (T = 168 h)	Trough Concentration prior to 3rd Dose (T = 336 h)	Trough Concentration Prior to 4th Dose (T = 672 h)
K1	23.31	27.72	45.61
K2	23.99	24.01	25.97
K3	25.14	26.74	24.21
K4	9.98	13.94	20.75
K5	30.88	17.67	25.93
K6	27.06	9.84	21.15
Mean (ng/mL)	23.39	19.99	27.27
SD (ng/mL)	7.11	7.30	9.27
Median (ng/mL)	24.56	20.84	25.07
Range (ng/mL)	9.98–30.88	9.84–27.72	20.75–45.61
Accumulation factor	1.26	1.33	1.35

## Data Availability

The data presented in this study are available in this article and the Supplementary Materials attached.

## Supplementary Materials

The following supporting information can be downloaded at: https://www.mdpi.com/2076-2615/12/3/250/s1. Table S1: Doxycycline plasma concentrations of the six koalas at various time point when administered doxycycline at 5 mg/kg via subcutaneous injection (diluted at 50:50 with saline), once a week for four weeks. Table S2: Pharmacokinetic parameters and indices of doxycycline in koala plasma determined over the first seven days after administration of doxycycline at 5 mg/kg via subcutaneous injection (diluted at 50:50 with saline). Table S3: Estimated doxycycline concentrations of the QC samples as triplicates dosed with doxycycline at different concentrations (125 and 1000 ng/mL), with the accuracy (%) and precision (%) of each intra-day.
