# Supplementary material for: Pharmacokinetic Profile of Doxycycline in Koala Plasma after Weekly Subcutaneous Injections for the Treatment of Chlamydiosis
Source: Animals (Basel). 2022 Jan 20;12(3):250. doi: 10.3390/ani12030250 (PMC8833767; doi:10.3390/ani12030250)
Supplement: Supplementary file 1 [file animals-12-00250-s001.zip › animals-1525522 supplementary material.pdf]

Supplementary Materials

Table S1. Doxycycline plasma concentrations of the six koalas in various time point when administered doxycycline 5 mg/kg by subcutaneous injection (diluted 50:50 with saline), once a week for four weeks.

	Doxycycline concentration in plasma (ng/mL)					
**T (h)**	**K1**	**K2**	**K3**	**K4**	**K5**	**K6**
0	0	0	0	0	0	0
1	283.45	288.25	170.77	138.38	328.89	498.89
2	296.41	265.53	264.36	167.40	236.17	474.38
4	213.07	377.40	244.85	140.53	238.78	408.68
8	166.01	312.56	262.23	124.47	209.26	310.98
12	116.06	268.88	245.74	104.41	191.21	323.39
24	107.64	224.18	168.99	64.09	155.07	237.03
48	84.75	N/A	121.07	47.23	107.49	140.61
72	N/A	71.18	N/A	N/A	N/A	N/A
96	45.78	N/A	94.87	12.70	48.70	74.84
120	N/A	39.25	N/A	N/A	N/A	N/A
144	26.18	N/A	23.68	24.02	15.10	36.38
168	23.31	23.99	25.14	9.98	30.88	27.06
336	27.72	24.01	26.74	13.94	17.67	9.84
504	22.95	33.38	11.40	10.42	15.67	21.76
672	45.61	25.97	24.21	20.75	25.93	21.15

N/A = not available.

Table S2. Pharmacokinetic parameters and indices of doxycycline in koala plasma determined over the first seven days after administration at doxycycline 5 mg/kg by subcutaneous injection (diluted with 50:50 saline).

	Two-compartmental analysis					One-compartmental analysis
**Parameters and indices**	**K1**	**K2**	**K3**	**K4**	**K6**	**K5**
K_{10} (1/h)	0.03	0.02	0.02	0.02	0.02	0.02
K_{12} (1/h)	0.12	0.01	0.01	0.02	0.03	-
K_{21} (1/h)	0.10	0.01	0.10	0.01	0.07	-
$T_{1/2}\ \alpha$ (h)	2.97	22.67	6.05	14.35	6.50	-
$T_{1/2}\ \beta$ (h)	64.25	117.89	46.02	137.87	48.61	-
$T_{1/2}\ K_{10}$ (h)	-	-	-	-	-	38.49
T_{max} (h)	0.20	3.06	4.01	2.14	0.27	0.42
C_{max} (ng/mL)	345.86	349.37	268.25	155.64	504.24	250.44

AUC_{0-t} (ng/mL*h)	11367.38	16733.25	15887.91	5725.13	20604.50	13330.46
$AUC_{0-\infty}$_obs (ng/mL*h)	13400.37	20594.33	17252.40	7765.74	22464.07	14011.32
AUC_{0-t} / $AUC_{0-\infty}$_obs	0.85	0.81	0.92	0.74	0.92	0.95
$AUMC_{0-\infty}$_obs (ng/mL*h^2)	1.16×10^6	2.08×10^6	1.14×10^6	1.01×10^6	1.45×10^6	7.79×10^5
MRT (h)	86.45	101.20	66.15	129.88	64.58	55.59
V_z/F _obs (L/kg)	14.03	13.19	16.65	29.37	9.77	19.82
Cl/F_obs (L/kg/h)	0.37	0.24	0.29	0.64	0.22	0.36

Table S3. Estimated doxycycline concentrations of the QC samples as triplicates dosed with doxycycline at different concentrations (125 and 1000 ng/mL), with the accuracy (%) and precision (%) of each intra-day.

	**Intra-day 1 (n = 3)**	
Expected concentration (ng/mL)	125	1000
Estimated concentration (ng/mL)	136.45 ± 12.74	1091.02 ± 82.76
Accuracy (%)	94.75 – 116.49	101.34 – 120.57
Precision (%)	9.33	7.59
	**Intra-day 2 (n = 3)**	
Expected concentration (ng/mL)	125	1000
Estimated concentration (ng/mL)	101.26 ± 2.75	1026.05 ± 67.90
Accuracy (%)	79.25 – 84.11	94.36 – 110.99
Precision (%)	2.71	6.62
	**Intra-day 3 (n = 3)**	
Expected concentration (ng/mL)	125	1000
Estimated concentration (ng/mL)	109.41 ± 4.48	1087.11 ± 68.77
Accuracy (%)	84.84 – 92.60	100.59 – 117.41
Precision (%)	4.1	6.33
